# Investigating the Link Between Specific Signaling Molecules (CCL5, CCR5, CXCR4, BMPR2) and Cardiac Health Indicators in Rheumatoid Arthritis Individuals: A Cross‐Sectional Study

**DOI:** 10.1002/hsr2.73232

**Published:** 2026-09-11

**Authors:** Nzar Hussein Hassan, Fatemeh Khoobbakht, Afsaneh Shamsi, Seyed Askar Roghani, Shirin Assar, Mehran Pournazari, Mahdi Taghadosi

**Affiliations:** ^1^ Immunology Department, Faculty of Medicine Kermanshah University of Medical Sciences Kermanshah Iran; ^2^ Student Research Committee Kermanshah University of Medical Sciences Kermanshah Iran; ^3^ Rheumatology research center, Tehran university of medical sciences, Tehran, iran Tehran University of Medical Sciences Tehran Iran; ^4^ Department of Immunology, School of Medicine Tehran University of Medical Sciences Tehran Iran; ^5^ Department of Anatomical Science, Faculty of Medical Science Tarbiat Modares University Tehran Iran; ^6^ Clinical Research Development Center, Imam Reza Hospital Kermanshah University of Medical Sciences Kermanshah Iran; ^7^ Cardiovascular Research Center, Health Technology Institute Kermanshah University of Medical Sciences Kermanshah Iran

**Keywords:** biomarkers, BMPR2, cardiovascular disease, CCL5, CCR5, CXCR4, HS‐CRP, inflammation, NT‐proBNP, rheumatoid arthritis

## Abstract

**Background and Aims:**

Persistent systemic inflammation is a hallmark of rheumatoid arthritis (RA) and contributes to increased cardiovascular disease (CVD) risk, a major cause of morbidity and mortality. Given the need for reliable biomarkers for cardiovascular risk stratification, this study aimed to evaluate the association between key signaling molecules (CCL5, CCR5, CXCR4, and BMPR2) and cardiovascular‐related biomarkers in RA patients.

**Methods:**

This cross‐sectional study was conducted between April and October 2022 and included 90 participants: 60 RA patients (30 treatment‐naïve and 30 receiving disease‐modifying anti‐rheumatic drugs) and 30 age‐ and sex‐matched healthy controls. Plasma CCL5 levels were measured using ELISA, and CCR5, CXCR4, and BMPR2 gene expression was assessed in peripheral blood mononuclear cells (PBMCs) using real‐time PCR and the Pfaffl method. Outcomes included high‐sensitivity C‐reactive protein (HS‐CRP), NT‐proBNP, disease activity (DAS‐28), and cardiovascular risk scores (FRS and SCORE). Group comparisons were performed using one‐way ANOVA with Tukey's post‐hoc test, and correlations were assessed using Pearson or Spearman methods as appropriate. Given the exploratory nature of the study, a Benjamini‐Hochberg false discovery rate (FDR) correction was additionally performed as a sensitivity analysis for multiple correlation testing.

**Results:**

Plasma CCL5 levels did not differ significantly among groups (*p* = 0.48). However, gene expression differed significantly for CCR5 (F[2,87] = 5.2, *p* = 0.006), CXCR4 (F[2,87] = 5.4, *p* = 0.005), and BMPR2 (F[2,87] = 3.9, *p* = 0.02), with higher expression in treatment‐naïve patients. Among RA patients (*n* = 60), CXCR4 expression correlated positively with DAS‐28 (*r* = 0.29, *p* = 0.02), HS‐CRP (*r* = 0.32, *p* = 0.01), and NT‐proBNP (*r* = 0.40, *p* = 0.002). Additionally, CCL5 (*r* = 0.39, *p* = 0.002) and CCR5 (*r* = 0.26, *p* = 0.046) showed positive correlations with NT‐proBNP. After FDR correction, the associations of CXCR4 with NT‐proBNP and HS‐CRP, and CCL5 with NT‐proBNP remained statistically significant, whereas borderline associations should be interpreted cautiously.

**Conclusion:**

CXCR4, CCL5, and CCR5 were associated with cardiovascular‐related biomarkers in RA patients. These findings provide preliminary evidence supporting further investigation of these molecules as potential biomarkers of cardiovascular involvement in RA. However, the observed associations are exploratory and observational in nature, do not imply causation, and require confirmation in larger prospective longitudinal studies before clinical risk‐stratification utility can be established.

## 1 Introduction

Rheumatoid arthritis (RA) is a chronic, systemic autoimmune disease affecting approximately 0.5%–1% of the global population. It is characterized by persistent synovial inflammation and progressive joint destruction, most commonly involving the small joints of the hands and feet, although larger joints such as the shoulders, elbows, and knees may also be affected [1, 2, 3, 4, 5].

Beyond articular manifestations, RA is increasingly recognized as a multisystem disorder, with involvement of various organs, including the cardiovascular, pulmonary, renal, neurological, ocular, and dermatological systems. Among these, cardiovascular disease (CVD) is one of the most important extra‐articular complications, significantly contributing to increased morbidity and premature mortality. Indeed, patients with RA have a 1.5‐ to twofold higher risk of developing CVD compared to the general population [6, 7, 8, 9, 10, 11, 12, 13].

Chronic systemic inflammation plays a central role in the pathogenesis of both RA and CVD, and several inflammatory mediators have been implicated in linking these conditions [14, 15]. The CXC chemokine receptor 4 (CXCR4), primarily expressed on naïve T cells, plays a critical role in cell trafficking, immune cell homing, and inflammatory responses. Its ligand, CXCL12, is produced by synovial endothelial cells and contributes to the recruitment and retention of inflammatory cells within the synovial tissue, thereby sustaining chronic inflammation [16, 17].

Similarly, the interaction between C‐C motif chemokine ligand 5 (CCL5) and its receptor CCR5 has been widely implicated in inflammatory conditions and chronic disease progression. CCL5 is produced predominantly by T lymphocytes, monocytes, and other immune cells, and it binds to several receptors, including CCR1, CCR3, CCR4, and CCR5, with CCR5 demonstrating the highest affinity. The CCL5–CCR5 axis plays a key role in leukocyte recruitment, activation, and amplification of inflammatory responses, thereby contributing to disease progression [18, 19, 20].

In addition, bone morphogenetic protein receptor type II (BMPR2), a member of the transforming growth factor‐β (TGF‐β) receptor family, plays an important role in vascular homeostasis and remodeling. Alterations in BMPR2 signaling have been associated with pulmonary arterial hypertension (PAH), a severe condition that can lead to heart failure and increased mortality, and has been reported in association with autoimmune diseases, including RA [21, 22].

Given the substantial burden of cardiovascular complications in RA patients and the growing interest in identifying reliable biomarkers for early cardiovascular risk detection, there is a need to better understand the role of inflammatory signaling pathways in mediating this association. Previous studies have highlighted the involvement of chemokines such as CXCL9 and CXCL12 in linking inflammation to cardiovascular outcomes; however, the role of CCL5, CCR5, CXCR4, and BMPR2 in this context remains insufficiently explored.

Therefore, the present study aimed to investigate the association between plasma CCL5 levels and the gene expression of CCR5, CXCR4, and BMPR2 (measured in peripheral blood mononuclear cells [PBMCs]) with key cardiovascular biomarkers, including high‐sensitivity C‐reactive protein (HS‐CRP) and N‐terminal pro‐B‐type natriuretic peptide (NT‐proBNP). Additionally, traditional cardiovascular risk factors and prediction tools, such as lipid profiles, body mass index (BMI), blood pressure, diabetes status, and established risk scores (FRS and SCORE), were evaluated to provide a more comprehensive assessment of cardiovascular risk in patients with RA.

## 2 Materials and Methods

### 2.1 Participant Cohort

This cross‐sectional study included 90 participants, consisting of 60 patients diagnosed with RA and 30 healthy control subjects matched for age and sex, which were recruited in our previous study [23]. Among the RA group, 30 participants were treatment‐naïve (newly diagnosed) and 30 were receiving standard anti‐rheumatic therapy, including disease‐modifying anti‐rheumatic drugs (DMARDs).

Participants were recruited from Imam Reza Hospital, affiliated with Kermanshah University of Medical Sciences (KUMS), between April 2022 and October 2022. The diagnosis of RA was established according to the 2010 American College of Rheumatology/European League Against Rheumatism (ACR/EULAR) classification criteria.

Exclusion criteria included a history of CVD, metabolic disorders (e.g., diabetes mellitus), malignancy, chronic liver or kidney disease, pregnancy, or other autoimmune/rheumatologic disorders.

To minimize potential confounding, control subjects were selected to match the RA group in terms of demographic characteristics. Potential sources of bias, including treatment‐related effects, were considered during the analysis.

Ethical approval was obtained from the Ethics Committee of KUMS (IR. KUMS. MED. REC.1401.292). All procedures were conducted in accordance with the Declaration of Helsinki, and written informed consent was obtained from all participants prior to enrollment. All participant data were anonymized and handled in accordance with institutional confidentiality policies.

### 2.2 Sample Collection and PBMC Isolation

A 5 mL sample of peripheral venous blood was collected from each participant into EDTA‐containing tubes.

Peripheral blood mononuclear cells (PBMCs) were isolated using density‐gradient centrifugation (e.g., Ficoll‐Paque method). The isolated PBMCs were used for RNA extraction and subsequent gene expression analysis.

Plasma was obtained by centrifugation at 3000 g for 10 min, and the supernatant was carefully aliquoted and stored at −80°C until further analysis.

RNA quality and purity were assessed using spectrophotometric analysis (A260/A280 ratio between 1.8 and 2.0 was considered acceptable).

### 2.3 ELISA for CCL5 Measurement

Plasma concentrations of CCL5 were measured using a commercially available ELISA kit (ZellBio GmbH, Germany; Catalog No: ZB‐13663C‐H9648), according to the manufacturer's instructions.

The assay detection range was 12.5–400 pg/mL.

All samples were analyzed in triplicate to ensure reliability, and the intra‐assay and inter‐assay coefficients of variation (CVs) were maintained below 10%.

### 2.4 Quantification of Gene Expression

Gene expression levels of CCR5, CXCR4, and BMPR2 were quantified using real‐time polymerase chain reaction (RT‐PCR) performed on a LightCycler 96 system (Roche Applied Science, Germany).

Complementary DNA (cDNA) was synthesized from extracted RNA, and amplification reactions were performed in a final volume of 15 μL, containing:

- 7.5 μL SYBR Green master mix (Ampliqon)
- 0.5 μL forward primer
- 0.5 μL reverse primer
- 1 μL cDNA template
- 5.5 μL nuclease‐free water

Thermal cycling conditions were as follows:

- Initial denaturation at 95°C for 30 s
- 40 amplification cycles (95°C for 5 s, 60°C for 30 s)
- Melting curve analysis to confirm specificity

All reactions were performed in triplicate. PCR efficiency ranged between 90% and 110%, fulfilling the assumptions required for Pfaffl analysis.

Relative gene expression was calculated using the Pfaffl method, normalized to the housekeeping gene GAPDH.

The primer sequences used for amplification are presented in Table 1.

**Table 1:** Primer sequences used for real‐time PCR analysis.

Product size (bp)	Primary sequence	Primer designation	Target gene	Type of gene
133	ACTACACCGAGGAAATGGGCT	CXCR4.F	CXCR4	Target genes
	CCCACAATGCCAGTTAAGAAGA	CXCR4.R		
75	CCGTTCCCCTACAAGAAACTC	CCR5.F	CCR5	
	GATGTCATAGATTGGACTTGACAC	CCR5.R		
120	GCTCGAAAGGTAGCACCTG	BMPR2.F	BMPR2	
	CACATTCTTCATAGTGACACTC	BMPR2.R		
188	GAAACCTGCCAAGTATGATG	GAPDH.F	GAPDH	Reference gene
	AGGAAATGAGCTTGACAAAG	GAPDH.R		

### 2.5 Evaluation of Cardiovascular Biomarkers and Risk Factors

- Cardiovascular risk assessment were evaluated in our previous works, including both laboratory biomarkers and clinical risk parameters [23, 24]:
- Fasting blood sugar (FBS)
- Lipid profile (total cholesterol, HDL, LDL, triglycerides)
- Thyroid function tests (T3, T4, TSH)
- High‐sensitivity C‐reactive protein (HS‐CRP)
- N‐terminal pro‐B‐type natriuretic peptide (NT‐proBNP)
- Serum HS‐CRP concentrations were measured using a high‐sensitivity immunoturbidimetric assay according to the manufacturer's instructions. NT‐proBNP levels were quantified using a commercially available electrochemiluminescence immunoassay (ECLIA). All laboratory analyses were performed in the central laboratory of Imam Reza Hospital under standardized quality‐control procedures.
- Additionally, cardiovascular risk was estimated using validated prediction models, including:
- Framingham Risk Score (FRS)
- Systematic Coronary Risk Evaluation (SCORE)
- The FRS was calculated using age, sex, systolic blood pressure, smoking status, total cholesterol, HDL cholesterol, and diabetes status. The SCORE risk estimation system was calculated according to the European Society of Cardiology recommendations using age, sex, smoking status, systolic blood pressure, and total cholesterol.

### 2.6 Statistical Analysis

Statistical analyses were performed using GraphPad Prism (version 6.0) and SPSS software (version 26.0, IBM, USA).

Data distribution was assessed using the Kolmogorov‐Smirnov test.

- Normally distributed variables were expressed as mean ± standard deviation (SD).
- Non‐normally distributed variables were presented as median and interquartile range (IQR).

Comparisons between groups were performed using one‐way analysis of variance (ANOVA), followed by Tukey's post‐hoc test to account for multiple comparisons.

Correlation analyses were conducted using:

- Pearson correlation for normally distributed variables.
- Spearman rank correlation for non‐parametric variables.

Because multiple correlation analyses were performed, the Benjamini‐Hochberg false discovery rate (FDR) procedure was applied as a sensitivity analysis to reduce the risk of type I error. Given the exploratory nature of this biomarker study, both unadjusted and FDR‐adjusted results were considered when interpreting findings.

To evaluate the potential influence of confounding variables, additional multivariable linear regression analyses were performed for selected outcomes. Models examining associations with NT‐proBNP were adjusted for age, sex, BMI, smoking status, disease duration, and treatment status.

The study was designed as an exploratory biomarker investigation. Because prior data regarding the expected associations between CCL5, CCR5, CXCR4, BMPR2, and cardiovascular biomarkers in RA patients were limited, a formal a priori sample‐size calculation was not performed. A post‐hoc assessment indicated that the available sample size provided adequate statistical power to detect moderate effect sizes.

All statistical tests were two‐sided, and a *p*‐value < 0.05 was considered statistically significant. Exact *p*‐values are reported. Correlation coefficients (r) are presented as measures of effect size.

Missing data were minimal (< 5%) and were handled using complete‐case analysis.

## 3 Results

### 3.1 Demographic and Clinical Characteristics

The study included a total of 90 participants, consisting of 30 treatment‐naïve RA patients, 30 RA patients receiving treatment, and 30 age‐ and sex‐matched healthy controls. There were no statistically significant differences among the three groups in terms of age or sex distribution (*p* > 0.05). However, disease duration was significantly longer in the on‐treatment group compared to treatment‐naïve patients (*p* < 0.01), as expected. No significant differences were observed in BMI or smoking status among the groups, indicating good comparability between study populations (Table 2).

**Table 2:** Demographic and clinical characteristics of the study participants.

Variable	Treatment‐Naïve RA (*n* = 30)	On‐treatment RA (*n* = 30)	Control (*n* = 30)	*p*‐value
Age (years), mean ± SD	48.80 ± 13.01	49.67 ± 10.51	48.10 ± 12.07	0.87
Female, *n* (%)	25 (83.3)	24 (80.0)	25 (83.3)	0.91
Disease duration (years), mean ± SD	0.8 ± 0.4	6.2 ± 3.1	—	< 0.01
BMI (kg/m^2^), mean ± SD	27.4 ± 4.2	26.8 ± 3.9	26.5 ± 3.6	0.68
Current Smoker, *n* (%)	6 (20.0)	5 (16.7)	4 (13.3)	0.74

*Note:* Data are expressed as mean ± SD or number (percentage). *p*‐values were calculated using one‐way ANOVA or chi‐square test as appropriate.

### 3.2 Plasma Levels of CCL5 and Participant Characteristics

The mean plasma concentration of CCL5 was 488.78 ± 211.44 pg/mL in treatment‐naïve patients, 425.83 ± 108.47 pg/mL in on‐treatment patients, and 519.84 ± 372.91 pg/mL in healthy controls. Analysis using one‐way ANOVA demonstrated that there were no statistically significant differences in CCL5 levels among the three groups (*p* = 0.48), indicating that circulating CCL5 concentrations were comparable regardless of disease status or treatment.

### 3.3 Gene Expression Analysis of CCR5, CXCR4, and BMPR2

**Figure 1:**
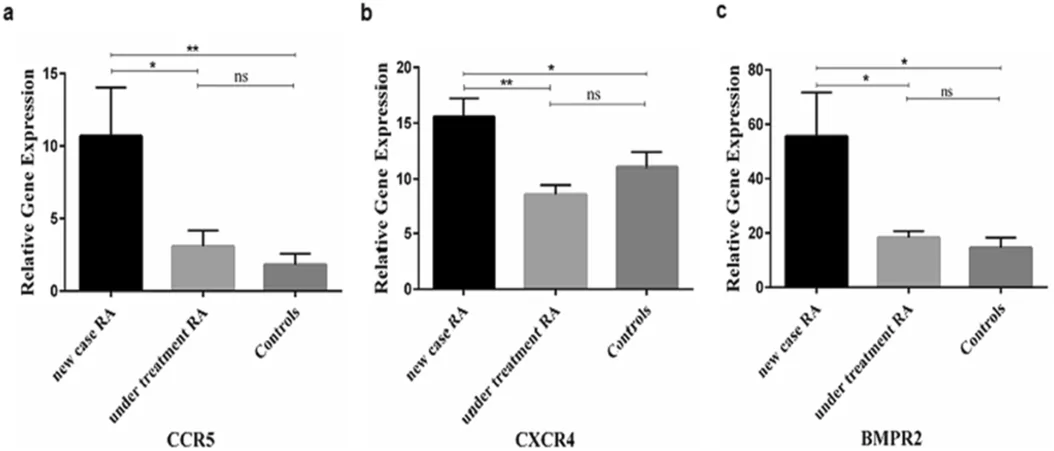
Comparison of relative gene expression levels of (a) CCR5, (b) CXCR4, and (c) BMPR2 among treatment‐naïve RA patients (*n* = 30), on‐treatment RA patients (*n* = 30), and healthy controls (*n* = 30). Gene expression was quantified in PBMCs by real‐time PCR and normalized to GAPDH using the Pfaffl method. Data are presented as mean ± SD. One‐way ANOVA followed by Tukey's post‐hoc test was used. *p < 0.05, **p < 0.01, ***p < 0.001 versus control; ^#^ *p* < 0.05, ^##^ *p* < 0.01 versus on‐treatment group.

The expression levels of CCR5, CXCR4, and BMPR2 genes differed significantly across the three groups (CCR5: F[2,87] = 5.2, *p* = 0.006; CXCR4: F[2,87] = 5.4, *p* = 0.005; BMPR2: F[2,87] = 3.9, *p* = 0.02; one‐way ANOVA) (Figure 1).

Post‐hoc analysis revealed that:

- CCR5 expression was significantly higher in treatment‐naïve RA patients compared to on‐treatment patients (*p* < 0.05) and controls (*p* < 0.01).
- CXCR4 expression was significantly elevated in treatment‐naïve patients compared to on‐treatment patients (*p* < 0.01) and controls (*p* < 0.05).
- BMPR2 expression was significantly higher in treatment‐naïve patients compared to both other groups (*p* < 0.01).

### 3.4 Correlation Analysis in RA Patients

**Figure 2:**
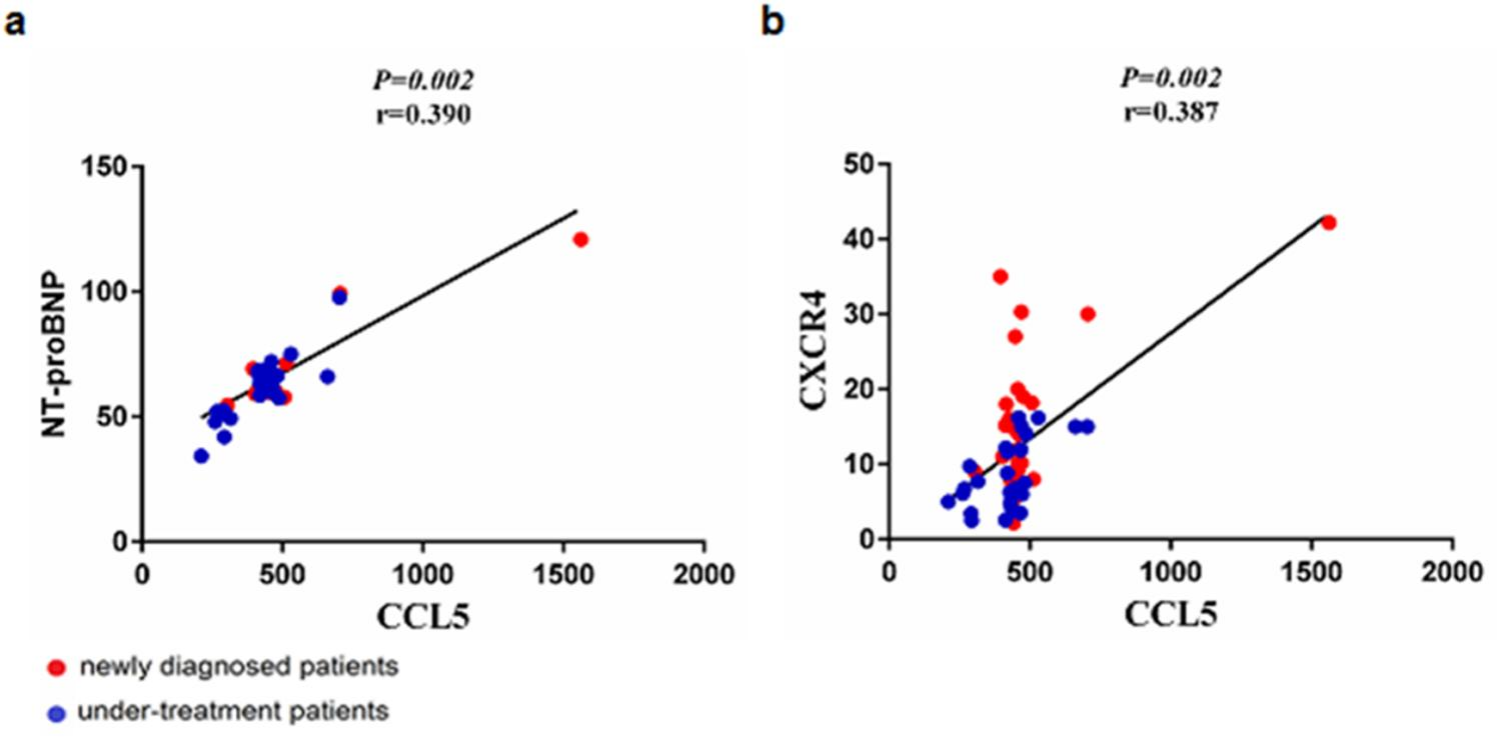
Spearman/Pearson correlation analysis between plasma CCL5 concentrations and (a) NT‐proBNP levels and (b) CXCR4 gene expression in RA patients (treatment‐naïve + on‐treatment, *n* = 60). The red point in panel (b) represents an outlier; sensitivity analysis confirmed the correlation remained significant after its removal (*r* = 0.387, *p* = 0.02). Data are shown with linear regression lines and 95% confidence intervals.

**Figure 3:**
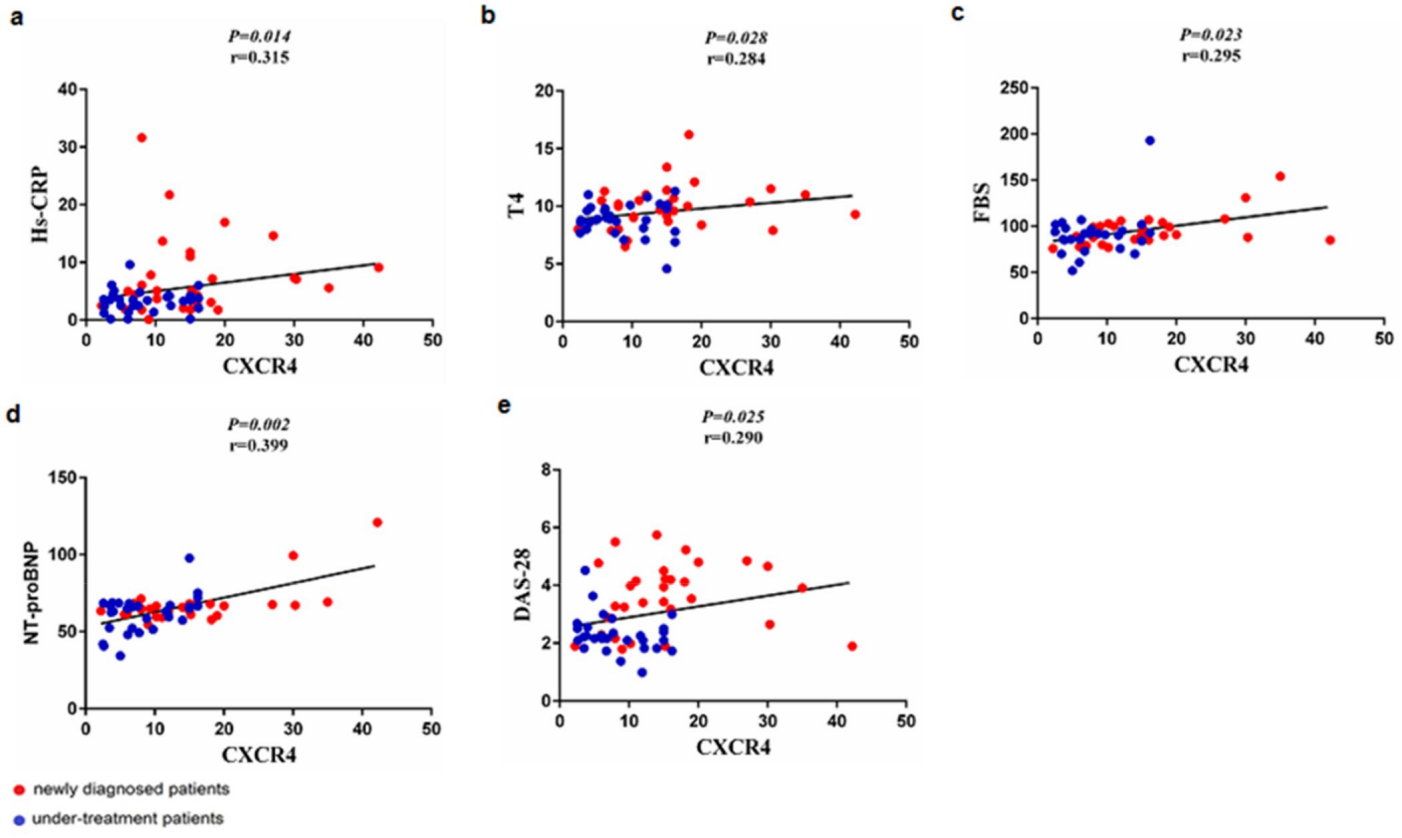
Correlations between CXCR4 gene expression and (a) HS‐CRP, (b) T4, (c) FBS, (d) NT‐proBNP, and (e) DAS‐28 in RA patients (*n* = 60). Pearson/Spearman correlation coefficients and *p*‐values are indicated in each panel. Data are presented with linear regression lines and 95% confidence intervals.

**Figure 4:**
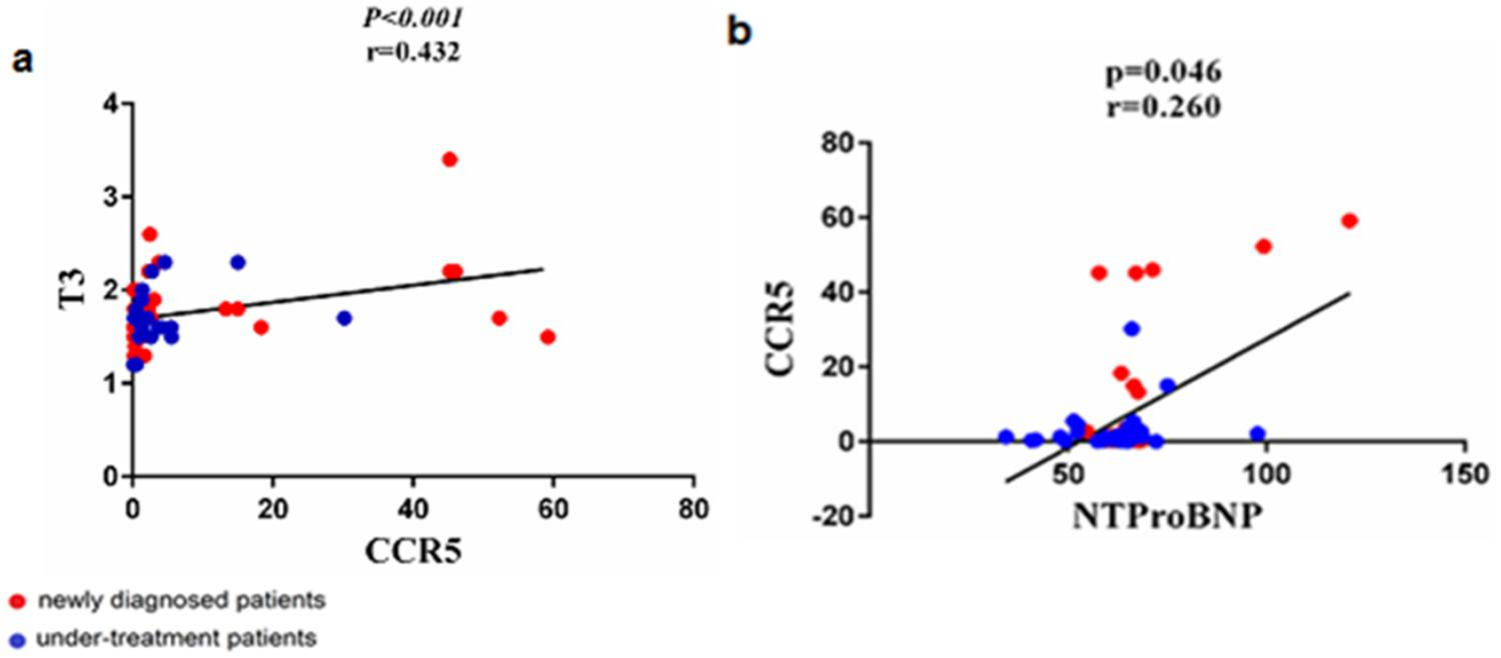
Correlations between CCR5 gene expression and (a) T3 and (b) NT‐proBNP levels in RA patients (*n* = 60). Data are shown with linear regression lines and 95% confidence intervals. Statistical analysis: Pearson/Spearman correlation.

**Figure 5:**
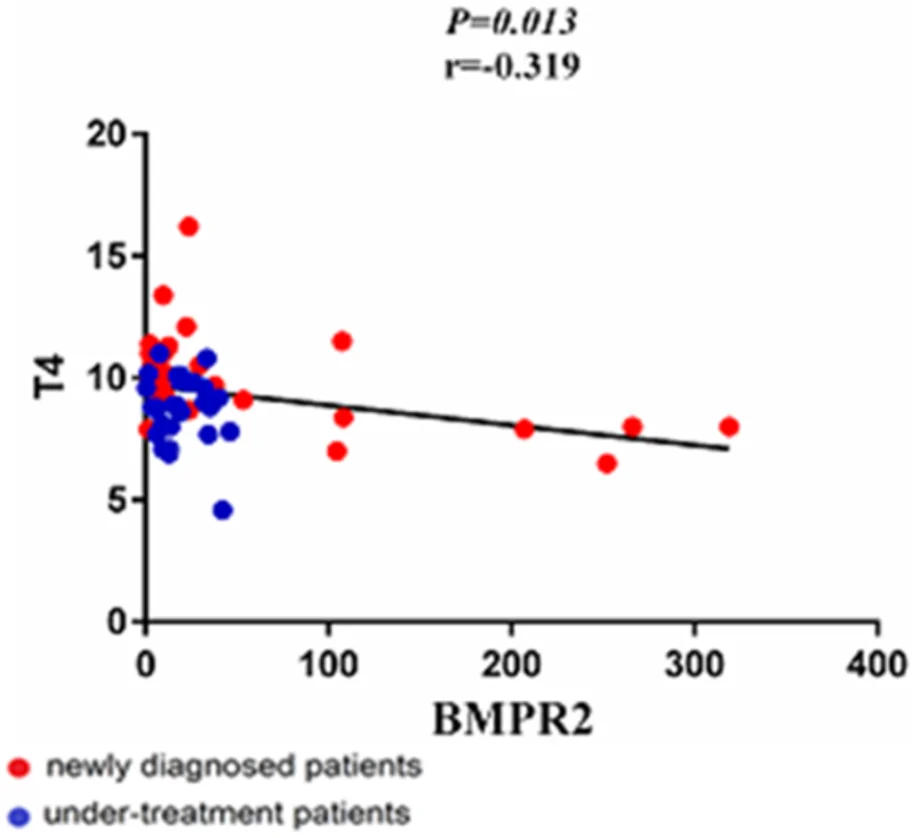
Negative correlation between BMPR2 gene expression and T4 levels in RA patients (*n* = 60). The linear regression line with 95% confidence interval is shown. Pearson correlation: *r* = −0.31, *p* = 0.01.

Correlation analyses were performed in the combined RA cohort (*n* = 60) to assess the relationship between molecular markers and clinical parameters. CXCR4 expression demonstrated significant positive correlations with disease activity and inflammatory markers, including DAS‐28 (*r* = 0.29, *p* = 0.02), HS‐CRP (*r* = 0.32, *p* = 0.01), and NT‐proBNP (*r* = 0.40, *p* = 0.002) (Figure 3). Furthermore, plasma CCL5 levels (*r* = 0.39, *p* = 0.002) and CCR5 expression (*r* = 0.26, *p* = 0.046) were also positively correlated with NT‐proBNP (Figures 2 and 4). These associations may indicate a relationship between inflammatory signaling pathways and cardiovascular stress in RA patients. No significant correlations were observed for BMPR2 expression with the clinical variables studied, except for a significant negative correlation with T4 levels (*r* = −0.32, *p* = 0.01) (Figure 5) (Table 3). To account for the relatively large number of correlation analyses performed, a Benjamini‐Hochberg FDR correction was applied as a sensitivity analysis. Following FDR adjustment, the associations between CXCR4 expression and NT‐proBNP, CXCR4 expression and HS‐CRP, CCL5 and NT‐proBNP, and CCR5 expression and T3 levels remained statistically significant. Associations with borderline significance in the unadjusted analyses, including the correlation between CCR5 and NT‐proBNP, did not consistently retain statistical significance after adjustment and should therefore be interpreted cautiously. Overall, the pattern of findings supported a relationship between inflammatory signaling pathways and cardiovascular stress‐related biomarkers, although these observations should be regarded as exploratory.

**Table 3:** Correlation analysis between molecular markers and clinical variables in RA patients.

Variable	CCL5 (r/*p*‐value)	CXCR4 (r/*p*‐value)	CCR5 (r/*p*‐value)	BMPR2 (r/*p*‐value)
DAS‐28	0.14/0.29	**0.29/0.02**	−0.03/0.81	−0.11/0.38
NT‐proBNP	**0.39/0.002**	**0.40/0.002**	**0.26/0.046**	0.02/0.91
FBS	−0.08/0.56	**0.30/0.02**	0.10/0.45	−0.07/0.62
HDL	−0.07/0.61	−0.22/0.09	−0.13/0.31	−0.20/0.13
LDL	−0.18/0.17	−0.19/0.14	0.18/0.16	−0.06/0.67
Cholesterol	−0.24/0.07	−0.10/0.43	0.16/0.21	0.02/0.90
TG	0.01/0.93	−0.01/0.96	0.09/0.48	−0.10/0.43
HS‐CRP	0.11/0.40	**0.32/0.01**	−0.02/0.90	−0.04/0.76
SCORE	0.14/0.31	0.11/0.41	0.06/0.66	0.03/0.83
FRS	0.07/0.59	0.01/0.92	0.12/0.38	0.04/0.79
T3	0.02/0.89	0.13/0.34	**0.43/** < **0.001**	−0.01/0.94
T4	0.10/0.45	**0.28/0.03**	0.12/0.37	**−0.32/0.01**

*Note:* Values are expressed as correlation coefficient (r) and *p*‐value. Significant associations (*p* < 0.05) are shown in bold. Pearson correlation was used for normally distributed variables and Spearman rank correlation for non‐normally distributed variables.

### 3.5 Outlier Analysis

Visual inspection of the scatter plots identified a potential outlier in the analysis of the association between CCL5 and CXCR4. A sensitivity analysis demonstrated that the observed correlation remained statistically significant after exclusion of this observation (*r* = 0.387, *p* = 0.02). Therefore, the association was not solely driven by a single extreme data point.

### 3.6 Multivariable Analysis

To evaluate the potential influence of confounding variables, multivariable linear regression analyses were performed using NT‐proBNP as the dependent variable. Models were adjusted for age, sex, BMI, smoking status, disease duration, and treatment status. The positive association between CXCR4 expression and NT‐proBNP remained significant after adjustment, suggesting that this relationship was not fully explained by the measured demographic and clinical factors. In contrast, weaker associations involving CCR5 and CCL5 were attenuated after adjustment and should be interpreted with caution. Despite these findings, residual confounding cannot be excluded because of the observational design and modest sample size.

## 4 Discussion

RA is a chronic inflammatory disorder strongly associated with an increased risk of CVD, largely driven by persistent systemic inflammation and immune dysregulation [6, 7, 8, 9, 10, 11, 12, 13]. In the present study, we investigated the relationship between key inflammatory signaling molecules (CCL5, CCR5, CXCR4, and BMPR2) and cardiovascular biomarkers in RA patients.

Our findings demonstrated that the gene expression levels of CCR5, CXCR4, and BMPR2 were significantly elevated in treatment‐naïve RA patients compared to both on‐treatment patients and healthy controls, whereas plasma CCL5 levels did not differ significantly between groups. These results suggest that gene expression changes may represent more sensitive indicators of inflammatory activity than circulating protein levels in this population. To our knowledge, this study is among the first to evaluate the combined role of the CCL5‐CCR5 axis, CXCR4 signaling, and BMPR2 expression in relation to cardiovascular biomarkers in RA patients.

The CCL5‐CCR5 axis has been widely implicated in inflammatory processes and atherosclerosis [18, 19, 20], with previous studies reporting increased CCL5 levels in serum and synovial fluid of RA patients [25, 26]. However, in contrast to prior findings, our study did not identify significant differences in plasma CCL5 levels among study groups, which may reflect variability in disease stage, sample characteristics, or treatment effects. Despite this, CCR5 expression was significantly elevated in treatment‐naïve patients, supporting its potential role in early immune activation and leukocyte recruitment, which are key mechanisms underlying both RA progression and vascular dysfunction.

The CXCR4/CXCL12 signaling axis plays a central role in immune cell trafficking and inflammatory responses [16, 27, 28]. In our study, CXCR4 expression was significantly increased in treatment‐naïve RA patients and showed positive correlations with multiple clinical parameters, including DAS‐28, HS‐CRP, NT‐proBNP, FBS, and T4.

Among all evaluated markers, the association between CXCR4 expression and NT‐proBNP was the most consistent finding. Furthermore, additional multivariable analyses adjusting for age, sex, BMI, smoking status, disease duration, and treatment status indicated that this relationship remained significant after adjustment. This observation suggests that the association was not entirely explained by the measured clinical and demographic factors, although residual confounding cannot be excluded.

Importantly, because multiple correlation analyses were conducted, the possibility of false‐positive findings must be considered. After application of the Benjamini‐Hochberg FDR correction, the associations of CXCR4 with NT‐proBNP and HS‐CRP, as well as the association between CCL5 and NT‐proBNP, remained statistically significant. In contrast, several weaker or borderline associations should be interpreted cautiously and regarded as exploratory.

Notably, the observed association between CXCR4 expression and NT‐proBNP (*r* = 0.40, *p* = 0.002) highlights a possible relationship between inflammatory signaling and cardiovascular stress in RA patients. NT‐proBNP is a well‐established biomarker of ventricular stress and heart failure and has been shown to increase in inflammatory conditions [29].

Nevertheless, the cross‐sectional design of the present study precludes the determination of temporal or causal relationships. Therefore, our findings should be interpreted as evidence of association rather than evidence that increased CXCR4 expression directly contributes to cardiovascular dysfunction.

Furthermore, the positive correlation between CXCR4 and HS‐CRP (*r* = 0.32, *p* = 0.01) reinforces the association between this receptor and systemic inflammation, which is a major contributor to atherosclerosis and cardiovascular risk in RA [30, 31]. These findings are consistent with previous studies indicating that the CXCL12/CXCR4 axis may be associated with adverse cardiovascular outcomes [32, 33, 34].

In addition, both CCL5 and CCR5 showed positive correlations with NT‐proBNP, suggesting that the CCL5‐CCR5 signaling pathway may be associated with cardiovascular alterations in RA patients. Although these associations were moderate in magnitude, they support the hypothesis that inflammatory chemokines may contribute to pathways involved in cardiovascular risk.

However, because some correlations were relatively weak and the study included a modest sample size, these findings should be interpreted with caution until replicated in larger independent cohorts.

In contrast, BMPR2 expression, although elevated in treatment‐naïve RA patients, did not show significant associations with key cardiovascular biomarkers such as NT‐proBNP and HS‐CRP. This finding differs from previous reports demonstrating an association between BMPR2 and CVD [35]. Such discrepancies may be attributed to differences in study design, sample size, or population characteristics, and further research is required to clarify this relationship.

An important consideration in this study is the potential confounding effect of treatment. Patients receiving anti‐rheumatic therapy exhibited lower expression levels of inflammatory genes, suggesting that pharmacological interventions, particularly DMARDs, may modulate inflammatory signaling pathways and influence cardiovascular biomarker levels. This highlights the importance of interpreting the results with caution, particularly in the context of treatment‐related effects.

This study has several limitations that should be acknowledged. First, the cross‐sectional design limits the ability to infer causality between inflammatory markers and cardiovascular outcomes. Second, the relatively small sample size may restrict the generalizability of the findings.

Although the study was adequately powered to detect moderate associations, it may have been underpowered to identify weaker correlations.

Third, although attempts were made to control for confounding variables and additional adjusted analyses were performed, residual confounding cannot be completely excluded.

Fourth, a relatively large number of correlations were examined. While an FDR correction was performed as a sensitivity analysis, the possibility of type I error cannot be entirely eliminated. Therefore, the results should be considered hypothesis‐generating.

Additionally, gene expression analysis was performed using PBMCs, which may not fully reflect tissue‐specific mechanisms involved in cardiovascular pathology.

Despite these limitations, this study has notable strengths, including the simultaneous evaluation of multiple inflammatory mediators, the comparison between treatment‐naïve and treated patients, the inclusion of cardiovascular biomarkers and risk scores, and the integrated assessment of inflammatory and cardiovascular parameters in the same patient cohort.

## 5 Conclusion

In conclusion, CXCR4, CCL5, and CCR5 were associated with cardiovascular‐related biomarkers in patients with RA. These findings suggest a potential relationship between inflammatory signaling pathways and cardiovascular stress in RA. However, due to the cross‐sectional design, modest sample size, and multiple comparisons performed, the results should be considered exploratory and interpreted cautiously. Further large‐scale prospective studies are needed to validate these findings and determine their potential clinical relevance.

## Author Contributions

**Nzar Hussein Hassan:** writing – original draft, writing – review and editing, investigation, resources. **Fatemeh Khoobbakht:** conceptualization, methodology, writing – review and editing, funding acquisition, project administration. **Afsaneh Shamsi:** writing – review and editing, investigation, resources. **Seyed Askar Roghani:** formal analysis. **Shirin Assar:** data curation. **Mehran Pournazari:** data curation. **Mahdi Taghadosi:** conceptualization, methodology, writing – review and editing, funding acquisition, project administration.

## Ethics Statement

The principles of the Helsinki Declaration were followed in the conduct of this investigation. Kermanshah University of Medical Sciences (KUMS), Kermanshah, Iran's Ethics Committee gave its approval (Ethical code: IR. KUMS. MED. REC.1401.292).

## Consent

The study obtained informed consent from each individual participant.

## Consent to Publish

The results of the study were published with the informed consent of human research participants, as confirmed by the authors.

## Conflicts of Interest

The authors declare no conflicts of interest.

## Transparency Statement

Mahdi Taghadosi, as the lead author, certifies the complete fidelity of this research report. This submission is founded on the principles of honesty, precision, and methodological clarity. We guarantee that all significant aspects of the investigation are included, and we have diligently accounted for and clarified any variance or departures from the initial study design.

## Data Availability

The data that support the findings of this study are available on request from the corresponding author. The data are not publicly available due to privacy or ethical restrictions. The datasets generated and/or analyzed during the current study are not publicly available due to ethical restrictions, but are available from the corresponding author upon reasonable request.
